# Population-Level Death Rates From Novel Coronavirus (COVID-19) in South Korea

**DOI:** 10.1177/1010539521993670

**Published:** 2021-02-09

**Authors:** Samir Soneji, Hiram Beltrán-Sánchez, JaeWon Yang, Caroline Mann

**Affiliations:** 1 University of North Carolina at Chapel Hill, NC, USA; 2 University of California, Los Angeles, CA, USA; 3 Brown University, Providence, RI, USA

## Introduction

In response to the rapidly growing number of coronavirus disease 2019 (COVID-19) cases, South Korea raised the national alert level to the highest possible level, instituted voluntary lockdown of affected cities and provinces, and implemented widespread screening.^1,2^ Case fatality rates (ratio of deaths to number infected) are the most common measure used to assess COVID-19 mortality burden. However, comparison of case fatality rates across countries is problematic because the true number of cases may far exceed the reported number of cases due to lack of testing.^3^ Additionally, COVID-19 is more fatal among older adults, and case fatality rates fail to account for age distribution that differ considerably between countries.^4-6^

To address this research gap, we utilize standard demographic and epidemiological principles to estimate the age-specific COVID-19 death rate in South Korea. These death rates can then be summarized into the age-standardized death rate (ASDR), which accounts for differences in population age distributions and allows appropriate comparison across countries.

## Methods

We began with the total number of COVID-19 cases and deaths by age group reported by South Korea to calculate case fatality rates and population-level prevalence.^5,7^ To estimate death rates by age, we fit a weighted logistic regression model using the penalized maximum likelihood method, which accounts for possible coefficient estimate bias from the small absolute number of deaths (especially among younger age groups).^8-10^ Estimated death rates were based on 207 days of exposure (January 21, 2020, to August 14, 2020). We annualized the estimated death rates by multiplying them by the inverse of the fraction of the year represented by the exposure period. We then calculated the ASDR for COVID-19, with the age standard based on the 2016 South Korean population (most recent year for which the ASDRs of the leading causes of death was published).^11^

We fit a similar weighted logistic regression model to estimate elasticities between (1) prevalence and death rates and (2) case fatality and death rates. Covariates were the natural logarithm of prevalence and natural logarithm of case fatality rates, both by age group. We considered alternative scenarios in which the (1) prevalence of COVID-19 increased across age groups and (2) COVID-19 death counts were underreported. For each scenario, we adjusted the estimated death rates by alternative prevalence rates and undercount percentages. We estimated the COVID-19 ASDR for each scenario and compared it with leading causes of death (see the appendix; available online).^11^

## Results

The first laboratory-confirmed COVID-19 case in South Korea occurred on January 21, 2020 (Supplemental Figure 1; available online). As of August 14, 2020 (207 days later), South Korea reported 14 873 cases (population-level prevalence of 0.03%) and 305 deaths. The case fatality rate increased from 0.1% among 30 to 39 year olds to 25.0% among ≥80 year olds.

**Figure 1.**
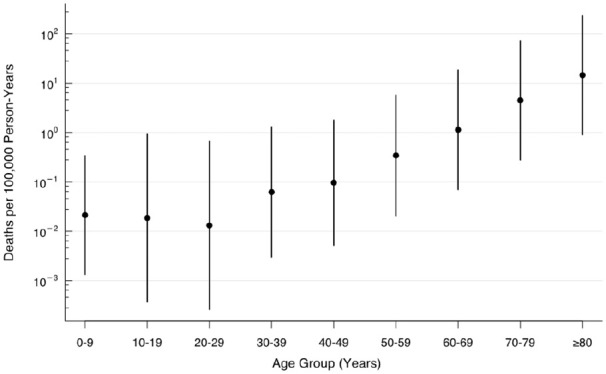
Population-level mortality rates for COVID-19, South Korea (as of May 20, 2020). Note: Vertical bars correspond to 95% confidence intervals estimated from a weighted logistic regression model using the penalized maximum likelihood method (see Methods section).

**Figure 2.**
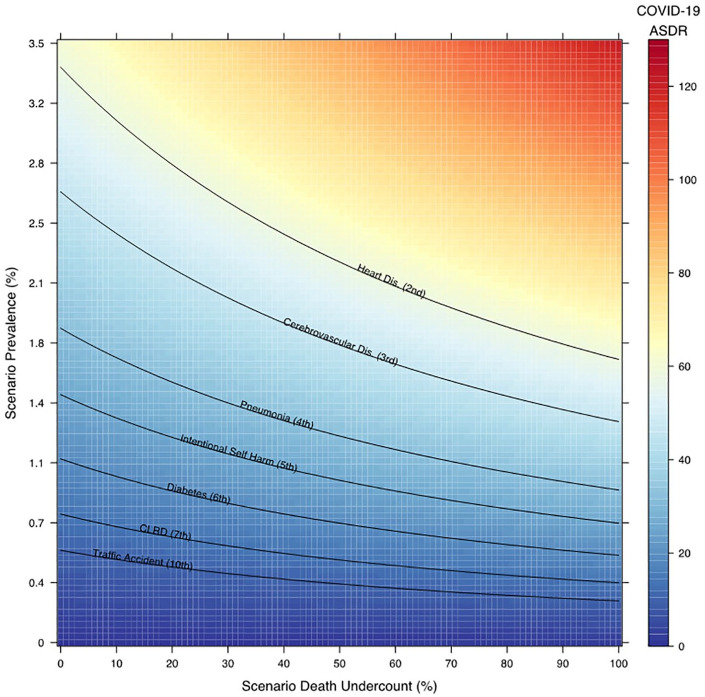
Age-standardized death rate of COVID-19 varying prevalence and undercount in deaths. Source: Authors’ calculation.

The estimated population-level death rate (deaths per 100 000 person-years) increased with age: 0.0 among 0 to 9 year olds, 0.1 among 40 to 49 year olds, and 14.5 among ≥80 year olds (Figure 1). The ASDR for COVID-19 was 0.9 deaths per 100 000 person-years and fell below the 10 leading causes of death (Figure 2). We estimated the elasticity between prevalence and deaths to equal 0.6; a 1% relative increase in the prevalence across all age groups was associated with a 0.6% increase in death counts and the death rate (Supplemental Table 2; available online).

The mortality burden of COVID-19, as measured by the ASDR, could increase substantially if either the number of COVID-19 deaths was underreported or the prevalence increases (Figure 2). If prevalence increased from 0.03% to 0.6%, the ASDR would increase from 0.9 to 10.0 deaths per 100 000 person-years and approximately equal that of traffic accidents to become the 10th leading cause of death. If the prevalence increased to 3.4%, the ASDR would increase to approximately the level of heart disease to become the second leading cause of death.

If the number of COVID-19 deaths has been underreported, smaller increases in the prevalence would lead to the disease becoming a leading cause of death. For example, if an additional 25% of COVID-19 deaths were not reported as decedents were not tested postmortem and prevalence increased from 0.03% to 0.6%, the ASDR would increase to the level of chronic lower respiratory tract disease (seventh leading cause of death). If the prevalence increased to 2.7%, the ASDR would increase to the level of heart disease (second leading cause of death).

## Discussion

The COVID-19 mortality burden depends on three broad factors: (1) the overall health of the population prior to the epidemic, (2) the prevalence of COVID-19, and (3) the lethality of COVID-19 infection. The second and third factors likely contributed more to the relatively low mortality burden in South Korea compared with countries with higher number of deaths (e.g., France, Italy, and Spain). With regard to the first factor, the overall health of the South Korean population was similar to that of the French, Italian, and Spanish populations prior to the pandemic, as measured by life expectancy at birth and ASDR from other respiratory diseases.^12^ With regard to the second factor, the prevalence of COVID-19 has remained comparatively low in South Korea because of successful containment and mitigation measures. These public health efforts have reduced the likelihood of transmission to individuals with comorbidities who are more likely than healthier individuals to be hospitalized, admitted to the intensive care unit, and die.^13^ With regard to the third broad factor, COVID-19 infection has also proven less lethal among the elderly in South Korea than in Italy. For example, the case fatality rate among ≥80 year olds equaled 25.9% in South Korea and 29.1% in Italy. However, rates could increase if the health care system becomes overwhelmed.^14^ Our approach enables researchers to estimate the COVID-19 ASDR based on country-specific prevalence and age-specific COVID-19 death rates, as well as account for differences in age distributions.

COVID-19 currently yields a relatively low mortality burden in South Korea compared with other causes of death because of its low prevalence. If the prevalence increases because of another outbreak or death counts have been underreported, the mortality burden could be substantially higher and exceed leading causes of death.

## Supplemental Material

sj-pdf-1-aph-10.1177_1010539521993670 – Supplemental material for Population-Level Death Rates From Novel Coronavirus (COVID-19) in South Korea Click here for additional data file. Supplemental material, sj-pdf-1-aph-10.1177_1010539521993670 for Population-Level Death Rates From Novel Coronavirus (COVID-19) in South Korea by Samir Soneji, Hiram Beltrán-Sánchez, JaeWon Yang and Caroline Mann in Asia Pacific Journal of Public Health
