# Supplementary material for: Population-Level Death Rates From Novel Coronavirus (COVID-19) in South Korea
Source: Asia Pac J Public Health. 2021 Feb 9;33(2-3):293–5. doi: 10.1177/1010539521993670 (PMC8142383; doi:10.1177/1010539521993670)
Supplement: sj-pdf-1-aph-10.1177_1010539521993670 – Supplemental material for Population-Level Death Rates From Novel Coronavirus (COVID-19) in South Korea [file sj-pdf-1-aph-10.1177_1010539521993670.pdf]

Supplement for "Population-Level Mortality Rates from Novel Coronavirus (COVID-19) in South Korea"

Samir Soneji* Hiram Beltrán-Sánchez† JaeWon Yang‡ Caroline Mann§

*Department of Health Behavior; University of North Carolina, Chapel Hill. Email: sonejis@email.unc.edu

†Department of Community Health Sciences and California Center for Population Research, University of California, Los Angeles. Email: beltrans@ucla.edu

‡Brown University

§University of North Carolina, Chapel Hill

A Confirmed Cases and Deaths from COVID-19, South Korea

Supplemental Figure 1: Number of Confirmed Cases of and Deaths from COVID-19, South Korea

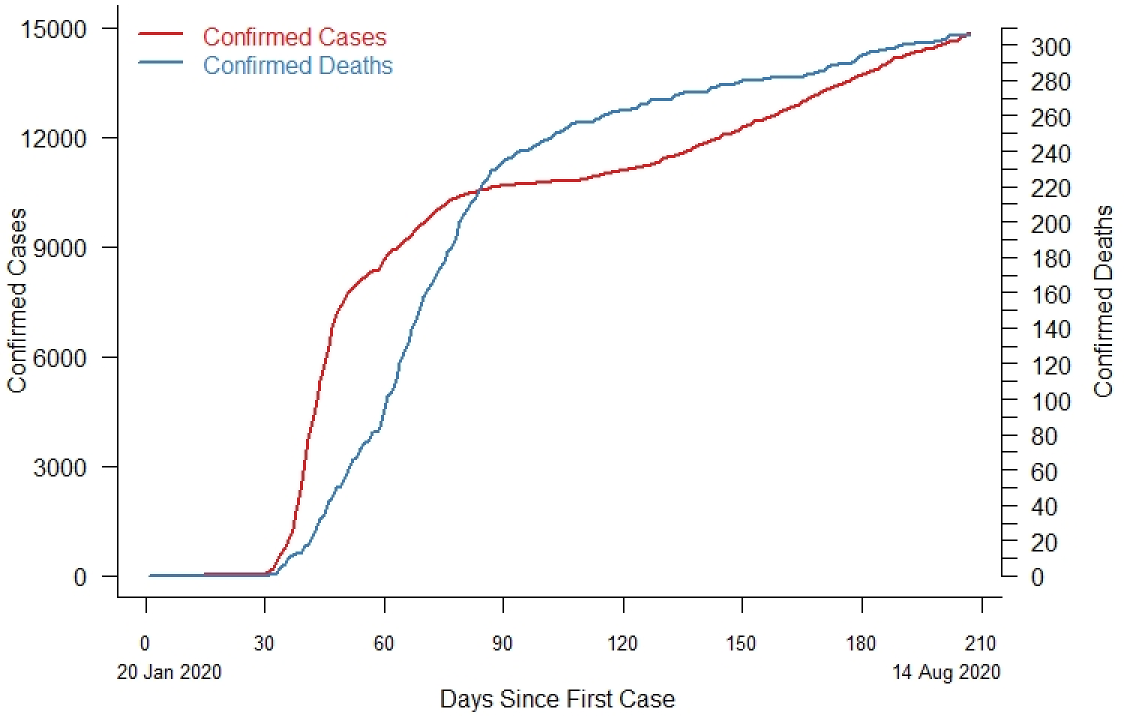

B Relationship Between Deaths, Case Fatality Rates, & Prevalence

Let *Deaths* represent the total number of reported deaths from the novel coronavirus (COVID-19) in a given country (e.g., South Korea) observed over a specified exposure period ($Exposure\ Period$). Let $\mathcal{A}$ be a set of the starting ages for the age intervals and ω represent the starting age of the oldest age interval. Let n_a represent the width (years) of an age interval starting at age $a \in \mathcal{A}$. Typically, the width of age intervals is the same for all but the oldest age interval $[\omega, \infty)$, that is $n_a = n$ for all $a \in \mathcal{A} \setminus \{\omega\}$ and $n_\omega = \infty$. Let $cases(a)$ represent the number of reported cases in age interval $a \in \mathcal{A}$.

$$Cases = \sum_{a \in \mathcal{A}} cases(a)$$

Similarly, let $deaths(a)$ represent the number of deaths in age interval $a \in \mathcal{A}$.

$$Deaths = \sum_{a \in \mathcal{A}} deaths(a)$$

Let $Case\,Fatality\,Rate$ represent the proportion of cases who died among all cases in age interval $a \in \mathcal{A}$.

$$Case\,Fatality\,Rate(a) = \frac{deaths(a)}{\sum_{a \in \mathcal{A}} cases(a)}$$

Let $N(a)$ represent the exposure measured in the number of person-years lived in the calendar year among individuals aged $[a, a + n_a)$. We approximate $N(a)$ by the mid-year population of age interval $a \in \mathcal{A}$. Finally, let $Prevalence(a)$ represent the population-level prevalence of COVID-19 in age interval $a \in \mathcal{A}$.

$$Prevalence(a) = \frac{cases(a)}{N(a)}$$

The population-level mortality rate, m, for age interval $a \in \mathcal{A}$ over the exposure period is equal to:

$$m(a) = \frac{deaths(a)}{N(a)}. \tag{1}$$

Thus, deaths can be expressed as a function of the mortality rate and the exposure (approximated by the mid-year population count).

$$deaths(a) = m(a) \times N(a) \tag{2}$$

We can express $m(a)$ in equation 1 as a function of the $Case\,Fatality\,Rate$ and $Prevalence(a)$ as follows.

$$\begin{aligned} m(a) &= \frac{deaths(a)}{N(a)} \\ &= \frac{deaths(a)}{N(a)} \times \frac{cases(a)}{cases(a)} \\ &= \frac{deaths(a)}{cases(a)} \times \frac{cases(a)}{N(a)} \\ &= Case\,Fatality\,Rate(a) \times Prevalence(a) \end{aligned} \tag{3}$$

Taking the natural logarithm of equation 3 and substituting $m(a)$ with its equivalent formulation in equation 1,

$$\begin{aligned} \log(m(a)) &= \log(Case\,Fatality\,Rate(a)) + \log(Prevalence(a)) \\ \log\left(\frac{deaths(a)}{N(a)}\right) &= \log\left(Case\,Fatality\,Rate(a)\right) + \log\left(Prevalence(a)\right) \\ \log\left(deaths(a)\right) &= \log\left(Case\,Fatality\,Rate(a)\right) + \log\left(Prevalence(a)\right) + \log\left(N(a)\right) \end{aligned} \tag{4}$$

C Estimated Population-Level Mortality Rates, South Korea

We fit a weighted logistic regression model using the penalized maximum likelihood method with weights equal to the exposure measured in person-years lived.[1–3] We utilized this method to account for possible bias from the small absolute number of COVID-19 deaths, especially among younger age groups. The outcome of the model was COVID-19 death and the covariate was age group. The estimated mortality rates were based on 85 days of exposure (21 January 2020 to 14 April 2020). To fit the weighted logistic regression model, we first transformed the death count data into long-form (Table 1). Table 3 shows the parameter estimates from the logistic regression.

We also fit a negative binomial regression and Poisson regression as alternative models. In both models, the outcome was the number of deaths by age group and the covariate was age group; the natural logarithm of the exposure was the offset. In all three models, the estimated mortality rates were based on 90 days of exposure (21 January 2020 to 19 April 2020). We annualized the estimated mortality rates by multiplying them by the inverse of the fraction of the year represented by the exposure period.

Figure 2 shows the estimated mortality rates for the three models. The estimated mortality rates for $\leq$ 29 year olds (i.e., the first three age groups) based on the negative binomial and Poisson models were substantially lower than the corresponding estimated mortality rates for these age groups based on the Firth weighted logistic model. Table 4 shows the estimated mortality rates and 95% confidence intervals by age group based on the Firth weighted logistic model.

Supplemental Table 1: COVID-19 Death Data, South Korea as of 14 August 2020 (Long-Form)

Age Category (Years)	Weight	COVID-19 Death (0 No, 1 Yes)
0-9	0	1
10-19	0	1
20-29	0	1
30-39	1	1
40-49	3	1
50-59	14	1
60-69	31	1
70-79	63	1
≥80	102	1
0-9	4,153,813	0
10-19	4,753,258	0
20-29	6,716,294	0
30-39	7,079,839	0
40-49	8,218,844	0
50-59	8,476,699	0
60-69	6,453,706	0
70-79	3,560,646	0
≥80	1,856,084	0

D Data Availability

All data and statistical code are freely available on the Harvard Dataverse repository.[4] The data are current as of 14 August 2020.

Supplemental Table 2: Estimated Elasticity Between Case Fatality Rates and Number of Deaths & Prevalence and Number of Deaths

	Point Estimate	Standard Error
Case Fatality Rates	0.17	0.02
Prevalence	0.56	0.11

Supplemental Table 3: Logistic Regression Results

	Point Estimate	Standard Error
Intercept	-15.93	1.41
Age Category (Ref: 0-9 Years)		
10-19	-0.13	2.00
20-29	-0.48	2.00
30-39	0.57	1.63
40-49	1.26	1.51
50-59	2.65	1.44
60-69	3.70	1.43
70-79	5.00	1.42
≥80	6.13	1.42

References

[1] Georg Heinze and Michael Schemper. A solution to the problem of separation in logistic regression. *Statistics in Medicine*, 21(16):2409–2419, 2002.

[2] David Firth. Bias reduction of maximum likelihood estimates. *Biometrika*, 80(1):27–38, 03 1993.

[3] Georg Heinze. A comparative investigation of methods for logistic regression with separated or nearly separated data. *Statistics in Medicine*, 25(24):4216–4226, 2006.

[4] Samir Soneji. Replication data for: Population-level mortality rates from novel coronavirus (covid-19) in south korea. Technical report, Harvard Dataverse, 2200.

Supplemental Figure 2: Estimated Mortality Rates by Age Group

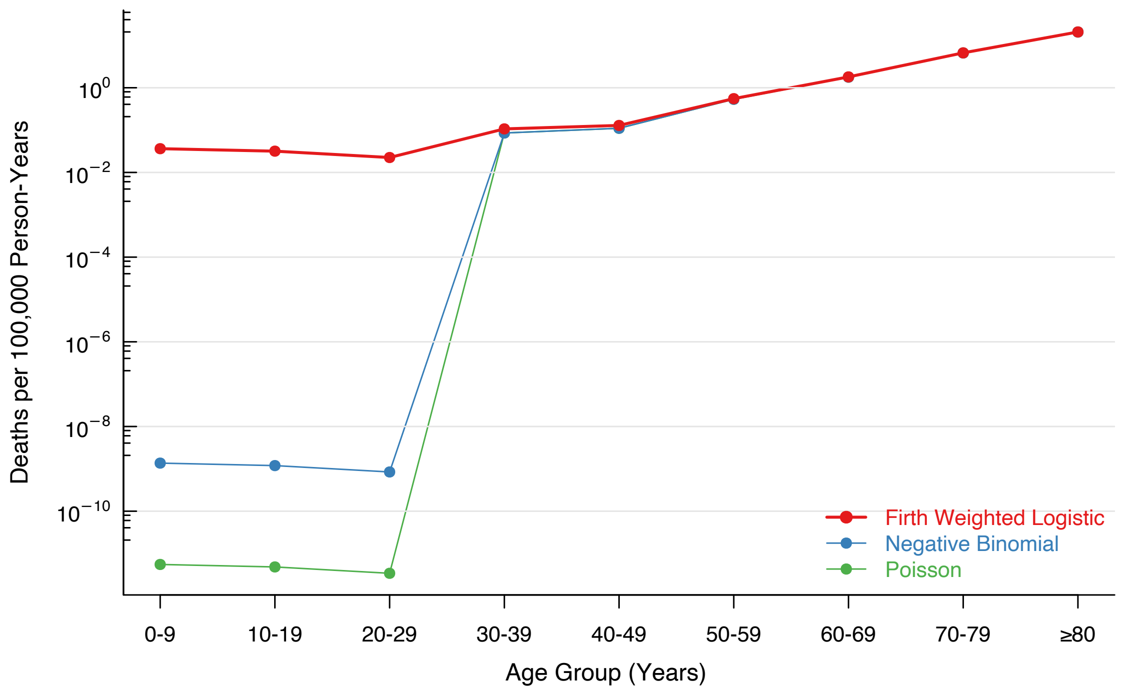

Supplemental Table 4: Population-Level Mortality Rates (Deaths per 100,000 Person-Years) Based on Firth Weighted Logistic Regression Model

Age Category (Years)	Pt. Est.	95% CI, Lower Bound	95%, Upper Bound
0-9	0.1	0.0	0.8
10-19	0.0	0.0	2.3
20-29	0.0	0.0	1.6
30-39	0.1	0.0	2.3
40-49	0.2	0.0	3.6
50-59	0.7	0.0	12.5
60-69	2.1	0.1	34.8
70-79	7.8	0.5	125.6
$\geq$ 80	24.1	1.5	387.3
